# Context counts! social anxiety modulates the processing of fearful faces in the context of chemosensory anxiety signals

**DOI:** 10.3389/fnhum.2013.00283

**Published:** 2013-06-19

**Authors:** Dirk Adolph, Lukas Meister, Bettina M. Pause

**Affiliations:** ^1^Department of Psychology, Ruhr-UniversityBochum, Germany; ^2^Department of Experimental Psychology, Heinrich-Heine-UniversityDüsseldorf, Germany

**Keywords:** chemosensory anxiety signals, context, emotion perception, emotion regulation, social anxiety, event-related-potentials, startle reaction

## Abstract

During emotion perception, context is an important source of information. Whether contextual cues from modalities other than vision or audition influence the perception of social emotional information has not been investigated. Thus, the present study aimed at testing emotion perception and regulation in response to fearful facial expressions presented in the context of chemosensory stimuli derived from sweat of anxious individuals. In groups of high (HSA) and low socially anxious (LSA) participants we recorded the startle reflex (Experiment I), and analysed event-related potentials (ERPs; Experiment II) while they viewed anxious facial expressions in the context of chemosensory anxiety signals and chemosensory control stimuli. Results revealed that N1/P1 and N170 amplitudes were larger while late positive potential (LPP) activity was smaller for facial expressions presented in the context of the anxiety and the chemosensory control stimulus as compared to facial expressions without a chemosensory context. Furthermore, HSA participants were highly sensitive to the contextual anxiety signals. They showed enhanced motivated attention allocation (LPP, Study II), as well as larger startle responses toward faces in the context of chemosensory anxiety signals than did LSA participants (Study I). Chemosensory context had no effect on emotion regulation, and both LSA and HSA participants showed effective emotion regulation (Study I and II). In conclusion, both anxiety and chemosensory sport context stimuli enhanced early attention allocation and structural encoding, but diminished motivated attention allocation to the facial expressions. The current results show that visual and chemosensory information is integrated on virtually all levels of stimulus processing and that socially anxious individuals might be especially sensitive to chemosensory contextual social information.

## Introduction

In social perception, context is an important source of information. For example, in everyday life the perception of a facial expression is almost always accompanied by a diverse range of contextual information, helping people to extract the social meaning of the situation (Aviezer et al., 2008). Accordingly, several studies have demonstrated that emotional context-information affects face perception and accompanying emotional responses (Kim et al., 2004; Schwarz et al., 2012). Although in everyday life a wide variety of contextual information is available, most studies investigating context effects uses visual or acoustic context information only. Whether also cues from other modalities influence the perception of emotional facial expressions has yet to be determined.

For example, chemosensory signals have been shown to modulate a wide variety of emotional responses, and its processing has been demonstrated to be largely independent of the allocation of attentional resources (Pause, 2012). Despite this, knowledge about their potency as context signals is rare. Thus, the main aim of the present study was to use chemosensory signals as context cues to investigate their impact on emotional responding toward facial expressions.

In detail, to investigate the potency to modulate emotional responding of the context stimulus, we measured defensive motivation during the perception of an anxiety related chemosensory context stimulus while viewing a fearful facial expression by means of the startle reflex (Experiment I). To elucidate the time course of central nervous processing event-related potentials (ERPs) in response to the face stimuli were analyzed (Experiment II). Because in everyday life people must often control their emotional responses to effectively adjust to the social environment (Gross et al., 2006), we investigated if the chemosensory context has an influence on the outcome of an emotion regulation task (Experiments I and II).

Emotion regulation refers to the extrinsic and intrinsic processes responsible for monitoring, evaluating, and modifying emotions (Thompson, 1994). One example is the cognitive reappraisal of emotion eliciting situations (Gross, 2002). Self-reported emotions, as well as physiological responses to threatening pictures including heart rate and electrodermal activity (Gross, 2002), brain electrical activity (Moser et al., 2009), neuronal responses in the amygdala (Ochsner et al., 2002), and the affect modulated startle-reflex (Jackson et al., 2000) can be significantly enhanced or reduced using cognitive reappraisal. To date, no study has tested whether chemosensory context information influences people's ability to regulate emotional responses.

However, beside emotion regulation, several studies indicate that, comparable to visual stimuli, also the delivery of social chemosensory information can alter emotional responses. For example, chemosensory signals of anxiety alter emotion related neuronal activity (Prehn-Kristensen et al., 2009) and enhance withdrawal related motivation (i.e., the startle reflex) in human perceivers (Prehn et al., 2006). Initial evidence suggests that chemosensory stimuli may also constitute powerful context cues for face perception (Li et al., 2007). Contextual chemosensory anxiety signals diminish the perceptual acuity of visual safety cues (happy facial expressions) (Pause et al., 2004), while the perceptual acuity of fear from ambiguous facial expression (morphs between happy and fearful facial expressions) is enhanced (Zhou and Chen, 2009). Moreover, motivated attention, as indicated by the late positive potential (LPP) within the ERP, directed toward neutral faces is enhanced when they are presented in the context of chemosensory stress signals (Rubin et al., 2012).

In social perception—including face perception and the perception of social chemosensory signals-social anxiety plays a modulating role. Social anxiety is characterized by abnormal processing of social threat information, involving processing biases in attending to, interpreting and remembering it (Hirsch and Clark, 2004). Accordingly, socially anxious individuals show deviant processing of single social fear relevant cues, including chemosensory anxiety signals, with examples being enhanced startle reactivity (Pause et al., 2009) and faster processing of chemosensory anxiety signals than non-anxious individuals (Pause et al., 2010). This suggests an attentional bias comparable to that observed with pictorial stimuli (see for example Kolassa and Miltner, 2006; Mühlberger et al., 2009).

Interestingly, social anxiety may also play a mediating role in the processing of threatening contextual information accompanying face perception, as it has been shown that threatening semantic information about a target facial expression enhances emotional responding in socially anxious individuals (Schwarz et al., 2012). Thus, converging evidence suggest that socially anxious individuals show sustained sensitivity toward various kinds of social signals, including chemosensory and visual signals of anxiety, as well as emotional context information. Therefore, in the present study, we compared a group of low socially anxious individuals (LSA) with a group of high socially anxious individuals (HSA).

### The present study

Two experiments assessed emotional reactivity and emotion regulation toward anxious facial expressions in the context of social chemosensory signals or control stimuli. The social chemosensory stimuli were chosen to be either congruent with the foreground picture stimulus (chemosensory anxiety signals derived from donors in an anxiety provoking situation) or incongruent with the facial stimulus (chemosensory exercise control stimuli). Furthermore, the anxiety signals were derived in a natural anxiety provoking situation, that is, the waiting for an oral examination at the university to reach an academic degree.

As dependent measures, the present study assessed the time course of stimulus processing including its early perceptual and attention sensitive (N1/P1), face specific (N170) and late motivational attention-related components (LPP), as well as the motivational/behavioral relevance of the stimuli (startle reflex). The startle reflex can be considered as a direct readout of activation of a defense system responsible to protect the organism from threat (see Bradley et al., 2001). The startle response is potentiated with the presence of a threatening stimulus (e.g., Bradley et al., 2001), and this potentiation has been argued to mirror the switch from orientation toward a meaningful stimulus to defense motivation as described by Sokolov (1963). In this light, startle potentiation can be characterized as the effect of motivational priming for action reflecting the defense system's general behavioral mobilization (Lang et al., 1997).

Study I assessed the motivational relevance of chemosensory context stimuli by withdrawal related motor behavior, emotional reactivity and emotion regulation using the startle reflex. Because startle responses elicited after target stimulus offset can reliably distinguish controls from phobics (Globisch et al., 1999), in the present study startle probes were elicited during and after the presentation of the target stimuli. If HSA participants are indeed especially sensitive to social stimuli, they should show sustained responsivity even after stimulus offset. Study II assessed the time course of stimulus processing with ERPs. In detail, an ERP component related to the early structural encoding of the stimuli (N1/P1, N170), as well as a component associated with the allocation of motivated attention (LPP) toward the stimuli were assessed. Previous research has verified that ERPs to emotional facial expressions are sensitive to the modulational effects of contextual information (e.g., the N170, see Righart and De Gelder, 2006, 2008).

We hypothesized that participants would show enhanced processing of faces in the context of chemosensory anxiety signals by showing larger startle amplitudes, enhanced early and late brain potentials, as well as less effective emotion regulation. We also predicted, that this effect would be most pronounced in socially anxious individuals.

## Experiment I

### Methods

#### Participants

Forty non-smoking female students participated in the study. They were recruited from the Heinrich-Heine-University of Düsseldorf, reported having a regular menstrual cycle, not using any medication including oral contraceptives and not suffering from mental and physical diseases. In addition none of them suffered from general hyposmia (three alternative forced choice test including one bottle with phenyl-ethyl-alcohol, 1:100, diluted in 1, 2-Propanediol, and two bottles containing the non-odorous solvent only). They were classified as either LSA (LSA, *n* = 20) or HSA (HSA, *n* = 20) based on their trait social anxiety scores (SIAS, Stangier et al., 1999). Participants scoring 22 or higher (>1.5 SD above the mean of the standard sample) were defined as HSA, those scoring 16 and lower (<0.5 SD above the mean of the standard sample) were defined as LSA. As a result, HSA participants scored well above the suggested cut-off score of 30 for social phobia (*M* = 34.05, *SD* = 9.12), while mean scores for the LSA group were within the normal range (*M* = 10.0, *SD* = 3.96; group comparison: *p* < 0.001). In contrast, HSA participants scored within the normal range on both trait anxiety (*M* = 42.05, *SD* = 9.12, STAI-X2, Laux et al., 1981) and self-reported depressive feelings [*M* = 8.51, *SD* = 5.66 Depressions Skala (DS); Von Zerssen and Koeller, 1976], while LSA participants scored low on both questionnaires (STAI: *M* = 35.60, *SD* = 4.50; DS: *M* = 3.75, *SD* = 2.45; group comparisons for both questionnaires: *p* < 0.01). Both groups scored within the medium range for empathy (LSA: *M* = 30.10, *SD* = 5.70; HSA: 29.20, *SD* = 5.37; Paulus, 2009), and the frequency of everyday-life use of reappraisal [LSA: *M* = 4.73, *SD* = 0.86; HSA: 4.93, *SD* = 0.73, Emotion Regulation Qestionnaire (ERQ); Abler and Kessler, 2009]. The two groups did not differ for age, *p* > 0.10 (*M* = 24.95, *SD* = 5.73, range 19–45). All participants were paid for their participation and gave written informed consent. The study was approved by the ethics committee of the German Psychological Society (DGPs).

#### Stimuli

***Chemosensory stimuli.*** To collect the chemosensory stimuli, axillary sweat was sampled from 20 male students of European descent from the University of Düsseldorf ^1^. All 20 donors donated sweat during both a natural anxiety provoking, and an exercise control condition (an important oral examination at the university, ergometer exercise). The donors' age ranged from 22 to 30 (*M* = 24.9, *SD* = 2.5). Their body mass index was within the normal range (range: 19.6-27.3, *M* = 23.2, *SD* = 1.9), and all reported to have a regular sleep-wake-cycle. All described themselves as healthy, especially with respect to hormonal, neurological, immunological, cardiological, and diseases of the axillae. They were within the normal range for trait anxiety (assessed with the STAI, *M* = 36.85, *SD* = 7.04). All participants donated sweat from both axillae for 90 min during an anxiety and a sport control donation situation using cotton pads (Ebelin Maxi Pads, dm-drugstore, Germany) while following a well-established sampling protocol (Pause et al., 2004, 2009; Prehn et al., 2006; Prehn-Kristensen et al., 2009). In detail, during an interview session, the donors gave written informed consent and were instructed to refrain from eating garlic, onions, asparagus, or spicy food, not to use deodorants and to wash their armpits exclusively with an unperfumed medical soap (Eubos®, Dr. Hobein GmbH, Germany) within 24 h prior to donation. In addition, to control for physiological arousal, the donors' heart rate was sampled during the interview session (baseline) and during the anxiety provoking and the sport control sampling condition using a mobile pulse monitor (R4 Plus, Omron, Germany). The anxiety condition consisted of 90 min of waiting for an important oral examination at the university in order to assess an academic degree (subjective importance, *M* = 8.29, *SD* = 0.87, scale range 0-10). Briefly before the donors entered the examination, they gave ratings of their current emotional state using the self-assessment manikin (SAM) (Bradley and Lang, 1994) (valence: −4 to 4, arousal: 1–9, dominance: 1–9), and the intensities of the six basic emotions (Ekman and Friesen, 1971) (10 cm visual analogue scales). In addition, the donors' heart rate was recorded. The sport control condition consisted of ergometer exercise and took place on average 6 (*SD* = 4.13) days after the anxiety condition, while the time of day was held constant (There was a mean difference of *M* = 83.75, *SD* = 85.65 minutes between the beginning of the anxiety and the sport control donation situation). To keep the physiological arousal comparable between the anxiety and the sport control condition, during ergometer exercise, donors' heart rate was held at the individual level that was recorded during the anxiety condition (using a mobile heart rate monitor, T 31, Polar, Germany). Briefly before the end of the sport control condition, the donors' emotional experience was assessed in the same way as in the anxiety condition.

During the anxiety condition, the donors described themselves as feeling more unpleasant, more aroused and less dominant (SAM ratings), as well as more anxious and less happy (basic emotions), than during the sport control condition (Table 1). There were no differences in ratings of disgust, sadness, surprise, or anger between the donation conditions. During the sport control condition the heart rate did not differ from the anxiety condition, *p* = 0.792 (anxiety condition: *M* = 91.25, *SD* = 22.07 beats per minute, sport control condition, *M* = 90.95, *SD* = 19.61 beats per minute). However, both heart rates were higher than during baseline recording (*M* = 68.80, *SD* = 11.22), both *p* < 0.001.

**Table 1:** **Sweat donors' self-reported intensities of basic emotions and SAM**.

	**Anxiety condition**	**Sport-control condition**	**Comparison**
	***M*_(*SD*)_**	***M*_(*SD*)_**	***P***
Anxiety	6.68_(1.69)_	0.46_(0.58)_	<0.001
Happiness	3.89_(2.37)_	7.09_(2.26)_	<0.001
Anger	1.74_(1.42)_	1.18_(1.54)_	n.s.
Disgust	0.90_(1.28)_	0.66_(1.23)_	n.s.
Sadness	1.78_(1.91)_	1.05_(1.06)_	n.s.
Surprise	3.04_(2.18)_	2.65_(2.45)_	n.s.
SAM_valence_	−0.05_(1.76)_	2.20_(1.28)_	<0.001
SAM_arousal_	7.35_(0.88)_	3.85_(1.60)_	<0.001
SAM_dominance_	4.50_(1.70)_	6.70_(1.31)_	<0.01

*Basic emotions range: 0–10 cm visual analogue scale, SAM_valence_ range −4–4, SAM_arousal_ and SAM_dominance_ range: 1–9*.

After all participants finished the sport control condition, the sweat samples were pooled with distinction to the respective donation condition (sport, anxiety) and stored at −20°C. The quantity of the sweat samples was largely the same for both condition (62 g for the sport control condition, 65 g for the anxiety condition). For the experiment, the homogenized samples were divided into small portions (1.2 g each).

***Visual stimuli.*** The visual stimulus material consisted of 14 pictures from 7 male actors (AM05, AM08, AM10, AM14, AM17, AM19, AM22) showing anxious facial expressions with averted gazes to the left and right and were selected from the Karolinska Directed Emotional Faces set (KDEF, Lundqvist et al., 1997) ^2^. These stimuli have been shown to reliably elicit emotional responses in women (Adolph and Alpers, 2010).

#### Stimulus presentation

The chemosensory stimuli were presented via a modified oxygen mask covering the nose and the mouth using a constant-flow (50 ml/s) 5-channel olfactometer (Lorig et al., 1999; Prehn-Kristensen et al., 2009) including five glass bottles. Two bottles were filled with 1.2 g of cotton pad (homogenized sweat samples either from the anxiety or the sport condition). The startle-eliciting stimulus was a 104 dB/A white noise burst (50 ms, rise-time <1 ms), presented through earplugs (ER4-14A Etymotic Research, USA), and calibrated using a high precision sound-level meter (Bruel & Kjaer, Denmark). Visual stimuli were shown on a 19″ monitor in a visual angle of 27 by 22°. Stimulus timing was controlled with the Presentation® software (Version14, Neurobehavioral Systems, USA).

#### Individual stimulus validation and odor detection session

For the present experiment participants needed to perceive the facial stimuli as fear inducing. This was tested in an individual session that took place no more than 7 days prior to the main experiment. During this session participants rated their emotional experience toward the pictures using the 6 basic emotions (10 cm visual analogue scales) and the valence and arousal scales of the SAM. As a result, for all participants, the most prominent reported emotion elicited by the pictures was anxiety (*M* = 5.53, *SD* = 1.66), differing significantly from the ratings of the other basic emotions (anger, disgust, sadness, happiness *p* < 0.01, surprise, *p* = 0.06). Consequently, no participants had to be excluded. Furthermore, participants rated their own emotional experience toward the pictures as negative (SAM valence, *M* = −1.13, *SD* = 1.01) and mildly arousing (SAM arousal *M* = 4.79, *SD* = 1.37). No differences emerged between HSA and LSA individuals in basic emotions or feelings according to the SAM-scale (all *p* > 0.10), with an exception for sadness [HSA > LSA, *t*_(38)_ = 2.13, *p* = 0.04].

During the same session participants also rated the chemosensory stimuli for intensity, pleasantness, unpleasantness, and familiarity (1 = not at all, 9 = extremely), and their own emotional experience toward the stimuli using the valence and arousal scales of the SAM. Results showed, that they perceived the stimuli as moderately intense (*M* = 5.35, *SD* = 1.68), unpleasant (*M* = 4.64, *SD* = 1.60), and familiar (*M* = 4.74, *SD* = 1.77), and as low in pleasantness (*M* = 3.19, *SD* = 1.43). Participants rated their own emotional response toward the chemosensory stimuli as mildly negative and mildly arousing (SAM valence: *M* = −0.64, *SD* = 1.15, SAM Arousal: *M* = 4.63, *SD* = 1.51). Ratings did not differ between chemosensory anxiety and sport stimuli (all *p* > 0.10) and between high and LSA individuals (all *p* > 0.10).

Of the participants twenty-six (65%) were able to differentiate the chemosensory stimuli from pure cotton pad (two correct detections for each stimulus within three-alternative forced choice tests including cotton pads from either condition, and two non-used cotton pads, all administered via the olfactometer for 2 s).

#### Experimental session

The experimental session was largely identical to that used in a previous study (Adolph and Pause, 2012). In brief, participants were seated in front of a computer screen, electrodes were attached and the order of stimulus delivery was explained. After participants signaled understanding of stimulus timing, they received detailed instructions on how to breathe during stimulus delivery. Inhalation was monitored using breathing belts (see Data Recording). The session did commence only, after the participants could control their breathing completely. Participants were then given detailed instructions to use cognitive linguistic emotion regulation strategies as used in our previous study. After verbatim instructions, participants were given two practice examples of how to accomplish emotion regulation in response to the face stimuli.

**Figure 1:**
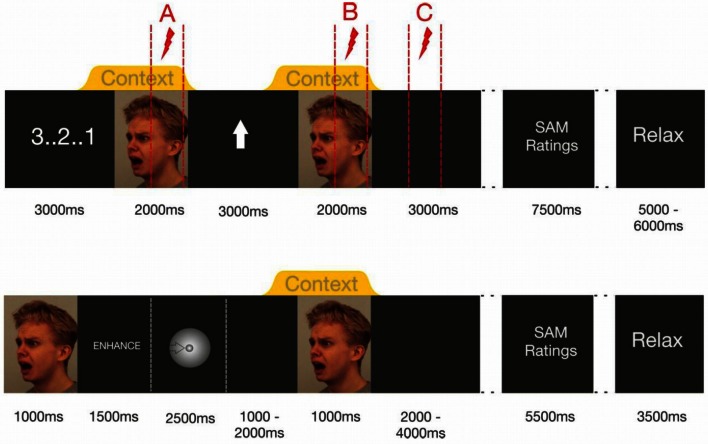
**Trial timing in Experiment I (upper half of figure) and Experiment II (lower half)**. Note that Experiment 1's dotted lines indicate the intervals in which startle probes were delivered. Within each trial only one of these probes was administered.

Upon providing verbatim emotion regulation instructions, participants received detailed instructions on how to use the emotion regulation strategies within the experimental protocol. Only after participants signaled full understanding of the emotion regulation procedure, they practiced at least 10 learning trials of each condition. The data recording began, when the participants reported successful emotion regulation in each condition and full compliance to the emotion regulation protocol. Upon start of the first emotion regulation block, participants received 8 startle probes to induce startle habituation. The visual and chemosensory stimuli were then presented in four blocks, counterbalancing the emotion regulation strategy and the chemosensory context (enhance/anxiety; enhance/sport; down-regulate/anxiety; down-regulate/sport) across participants. Each block consisted of 21 trials, with 10-min breaks between the blocks. Figure 1 shows the sequence of stimulus presentations.

At the beginning of each trial, a visual countdown was presented, followed by the presentation of a fearful facial expression (2000 ms baseline stimulus), in the context of either a chemosensory anxiety signal, or a chemosensory sport stimulus, beginning 500 ms before the onset of and co-terminating with the face stimulus. Then, a visual regulation cue was presented for 3000 ms, an upward-pointing arrow signaling “enhance,” or a downward-pointing arrow signaling “down-regulate.” The same picture-chemosensory context combination was then presented again (target stimulus). Then, a black screen (3000 ms) followed by the valence and arousal scales of the SAM (3500 ms each with a 500 ms break between the scales) was presented. Thereafter, the participants were free to relax for an interval varying randomly between 5 and 6 s. To prevent habituation effects, two identical chemosensory stimuli never followed each other in consecutive trials. All 7 picture stimuli were delivered to all participants, and the sequence of stimuli was randomized across trials within one block of stimulus presentations.

During each trial one acoustic startle probe was presented to assess emotional responding. Shown in Figure 1 (upper half) is that probes could occur at three different positions during the trials (Probe A = baseline: 1000–1900 ms after onset of the baseline stimulus, Probe B = target picture perception: 1000–1900 ms after the onset of the target stimulus, Probe C = target picture offset: 2000–2900 ms after the offset of the target stimulus) [see Figure 1 (upper half)]. During each block, seven startle probes were presented at each probe position resulting in 21 startle-probes per block (enhance anxiety, enhance sport control, down-regulate anxiety, down-regulate sport control). Trials including different startle-probe positions were equally distributed within the blocks. Each stimulus was probed once at each probe position (A, B, C) in each block.

After the last emotion regulation block had ended participants completed a questionnaire in which they were asked to freely describe the strategies they used to enhance and down-regulate their emotions.

#### Data recording

The startle eyeblink was recorded from the orbicularis oculi muscle beneath the left eye using two Ag/AgCl electrodes (inner diameter 5 mm). Participants breathing cycles were assessed with two respiration belts (Brain Products, Germany) placed around participants' abdomen and thorax. The physiological data were amplified (22bit Quick-Amp, Brain Products, Germany) and recorded with BrainVision Recorder Software (Brain Products, Germany), sampled at 2000 Hz, and filtered on-line using a 50 Hz notch filter. Off-line, the raw EMG was high- (28 Hz, 24 dB/octave) and low-pass filtered (500 Hz, 24 dB/octave) (Van Boxtel et al., 1998).

#### Data reduction

Of the eyeblinkresponses 3.3% were rejected because they were recorded neither during an increase in inhalation nor briefly (200 ms) after the inhalation maximum, 1.0% because the blink onset occurred during baseline. The remaining trials were rectified and smoothed (20 ms moving average). The startle data were baseline corrected (0–20 ms after startle probe onset), and the startle-response was scored as the maximum deflection within 30–150 ms after startle probe onset. Non-responses (amplitudes ≤ 2 × the largest amplitude within the baseline interval; 1.2% responses) were scored as 0. Outlier values differing more than two standard deviations from the condition average were excluded (1.7% of responses) (Blumenthal et al., 2005). Due to excessive differences in startle amplitude the startle-responses were *z*-standardized within each participant and across conditions.

#### Data analysis

For each picture-chemosensory stimulus combination, the baseline emotional response was assessed as the response to Startle-Probe A within each of the four experimental block. In addition, the regulated emotional response was assessed as the response to startle probes B and C, resulting in one baseline emotional response, and two regulated emotional responses for each of the four blocks. Thus, analysis of variance (ANOVA) including one between subject factor, 2 (*Group*: HSA, LSA) and the within subjective factors, 2 (*Context*: anxiety, sport control) × 2 (*Emotion regulation*: enhance, down-regulate) × 3 (*Time*: A = baseline, B = target picture perception, C = target picture offset) were run. Statistical analyses were performed using SPSS 18, and Cohen's effect-size *f* was calculated. Huynh–Feldt corrections of degrees of freedom were applied, and corrected *p*-values are reported. Subsequent nested effects (Page et al., 2003) and *t*-tests were calculated. An alpha level of 5% was used for all statistical tests.

For SAM ratings 2 (*Group*) × 2 (*Context*) × 2 (*Emotion Regulation*) ANOVAs were run. Cohen's effect-size *f* was calculated, Huynh–Feldt corrections were applied, and corrected *p*-values are reported.

### Results

#### Emotion regulation strategies

The individual answers of the 40 participants to the post experimental questionnaire on emotion regulation strategies initially were classified by two independent raters. Overall agreement was high between the raters on the single emotion regulation strategies. To down-regulate, the majority of participants reported rationalizing or reinterpreting the expression (*N* = 17, 42.5% of all participants). For example, participants reported imagining the face as a comic strip or a photo, or imagined that the expression was triggered by something not dangerous. Most of the remaining participants reported focusing on possible positive aspects or outcome of an imagined situation corresponding to the expression (*N* = 16, 40% of all participants). For example, they imagined an assault but that the offender was arrested. Seven participants (3.8%) reported to use strategies other than these two. For example, that they were just trying to keep detached from the person on the photo.

To enhance their emotional response, the majority of participants tried to feel what the person feels on the photo (*N* = 24, 60% of all participants). Most of the remaining participants reported focusing on negative aspects or outcome of an imagined situation for the person on the photo (*N* = 14, 35% of all participants). For example, participants reported imagining being the victim of an assault together with the person on the photo. A total of 2 participants (5%) reported the use of other strategies, for example, to simply concentrate more on the respective expression. Results of χ^2^ tests indicate that the frequency of use of the different regulation strategies did not differ between HSA and LSA participants (all *p* > 0.10).

#### Effects of chemosensory context

***Ratings.*** Independent of the emotion regulation strategy, the participants felt more negative when the faces were presented along with a chemosensory anxiety cue, than with the chemosensory sport cue, *F*_(1, 38)_ = 8.07, *p* = 0.007, *f* = 0.46 (Main effect for *Context*) (Table 2).

**Table 2:** **Emotion regulation effects on subjective ratings and the startle reflex toward anxious faces in the context of chemosensory anxiety or sport-control stimuli in Experiment I (Startle)**.

	**Chemosensory sport control**		**Chemosensory anxiety**	
	**Enhance**	**Down-regulate**	**Enhance**	**Down-regulate**
	***M*_(*SD*)_**	***M*_(*SD*)_**	***M*_(*SD*)_**	***M*_(*SD*)_**
SAM_valence_	−1.70_(0.78)_	−0.36_(1.10)_	−1.80_(0.72)_	−0.45_(1.08)_
SAM_arousal_	6.14_(1.20)_	4.28_(1.36)_	6.16_(1.20)_	4.42_(1.39)_
Startle_probeA_	0.05_(1.20)_	−0.22_(0.56)_	0.10_(0,68)_	0.01_(0,65)_
Startle_probeB_	0.11_(0.60)_	−0.09_(0.61)_	0.05_(0.57)_	−0.10_(0.51)_
Startle_probeC_	0.24_(0.74)_	−0.19_(0.61)_	0.13_(0.59)_	−0.08_(0.54)_

*SAM_valence_ range: −4–4; SAM_arousal_ range: 1–9, Startle responses are given as z-score*.

**Figure 2:**
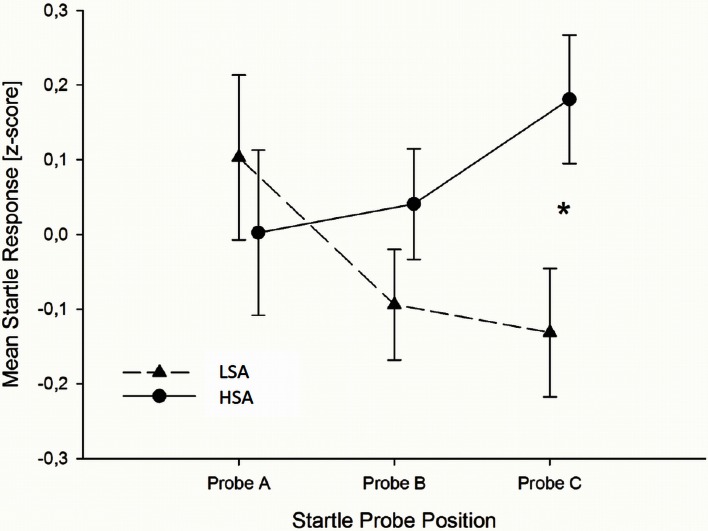
**In Experiment I, HSA participants (dashed lines) showed larger startle magnitudes than LSA participants (sold lines) toward the anxious facial expression presented in the context of the chemosensory anxiety signal during startle position C**. Furthermore, for HSA participants startle responses did not differ between startle probes A, B, and C, while for LSA participants startle responses were smaller during startle probe B, and probe C, than probe A, indicating startle habituation in LSA, but not in HSA participants. ^*^0.040.

***Startle reflex.*** Independently of the emotion regulation strategy, HSA individuals showed larger startle magnitudes toward the faces presented in the context of chemosensory anxiety cues than the LSA participants, especially toward startles presented at probe position C, *F*_(2, 76)_ = 3.36, *p* = 0.040, *f* = 0.30 [Interaction *Context* by *Time* by *Group*, nested effects: *Group* by *Context* within probe C: *F*_(1, 38)_ = 4.69, *p* = 0.037; *Group* within probe C within HSA: *F*_(1, 38)_ = 6.60, *p* = 0.014] [Figure 2]. No differences between HSA and LSA participants occurred for faces presented in the context of chemosensory sport control stimuli (*p* > 0.10).

To further explore the differences in startle responses toward faces presented in the context of anxiety signals, we calculated habituation of startle responses for HSA and LSA individuals. Results show, that for LSA individuals startle responses elicited during the anxiety context habituated rapidly within the trials. In detail, startle responses elicited during baseline stimuli perception (probe A) were larger than those elicited during target stimulus perception (probe B), *t*_(19)_ = 2.022, *p* = 0.029, one-tailed, and larger than those elicited during the late emotion regulation interval (probe C), *t*_(19)_ = 1.794, *p* = 0.045, one-tailed. For socially anxious individuals startle responses elicited during the anxiety context did not habituate. That is, startle responses do not differ between baseline, target stimulus perception and late emotion regulation interval, all *p* > 0.10, one-tailed (see Figure 2).

#### Effects of emotion regulation strategies

***Self-Ratings.*** After down-regulating their emotions, participants rated their own emotional experience as neutral (Table 2) and significantly less negative than after enhancing their emotion, *F*_(1, 38)_ = 57.60, *p* < 0.001, *f* = 1.23 (Main effect for *Emotion Regulation*). Similarly, after down-regulating, participants rated their experienced arousal as neutral and significantly lower, as after enhancing their emotion, *F*_(1, 38)_ = 68.61, *p* < 0.001, *f* = 1.34 (Main effect for *Emotion Regulation*). There were no more significant ANOVA effects concerning the ratings (all *p* > 0.10).

***Startle-reflex.*** Regardless of *Context* and *Group*, participants exhibited smaller startle magnitudes toward probes in the down-regulation (*M* = −0.112, *SD* = 0.288) as compared to the enhance condition (*M* = 0.112, *SD* = 0.288), *F*_(1, 38)_ = 5.99, *p* = 0.019, *f* = 0.40 (Main effect for *Emotion Regulation*) suggesting successful emotion regulation. To clarify whether this significant differences between the enhance- and the down-regulate condition was due to successful enhancement or successful down-regulation of emotions, the startle responses during emotion regulation were compared to the baseline responses (collapsed over *Context*, as well as early and late emotion regulation interval). These analysis show that enhancement of emotions was successful, *t*_(39)_ = 1.98, *p* = 0.027, *d* = 0.32 (one-tailed), and that down-regulation of emotions tended to be effective, *t*_(39)_ = −1.52, *p* = 0.069, *d* = 0.25 (one-tailed).

### Discussion

In Experiment I, emotion perception and regulation was assessed in response to anxious facial expressions presented in the context of chemosensory anxiety signals. In line with the hypothesis, when presented in the context of chemosensory anxiety signals, the faces were rated as more negative than when presented in the context of chemosensory sport stimuli. Moreover, when a startle response was elicited during face presentations in the chemosensory anxiety context, it was elevated in HSA participants compared to LSA participants. These results show that chemosensory anxiety related context information is capable of altering behaviorally relevant emotional responses (i.e., withdrawal related motor behavior and self-report) toward socially relevant visual stimuli. Thus, the results are in line with findings of altered visual social perception through chemosensory anxiety signals (Pause et al., 2004; Zhou and Chen, 2009). The fact that the effect of anxiety relevant chemosensory context stimuli was especially pronounced in HSA individuals extends previous work showing a hyperreactivity of HSA individuals toward facial (Blair et al., 2008) and chemosensory signals of anxiety (for a comprehensive discussion see Pause et al., 2009). Furthermore, the results are in line with previous research showing that neutral faces presented in a negative self-evaluative semantic context affects neural responses in HSA individuals more strongly than in healthy controls (Schwarz et al., 2012). The present data extend these results and show for the first time that socially anxious individuals might be especially prone to the impact of non-semantic (threatening) chemosensory context information on the perception of emotional faces. Furthermore, the fact that startle responses differentiate between HSA and LSA individuals mainly in response to startle probe C, that is briefly after target stimulus offset, further underlines the sensitivity of socially anxious individuals toward even weak social cues.

Both, HSA and LSA participants were able to effectively regulate their emotions toward the faces: they exhibited smaller startle-responses, felt less negative and less aroused when down regulating, than when enhancing their emotions. Thus, while emotion regulation toward social cues in HSA participants has been demonstrated before (Goldin et al., 2009), the present results show that also defensive motivation toward socially relevant stimuli can be regulated effectively. Thus, chemosensory context had no influence on the participants' ability to regulate their emotions. Contextual chemosensory anxiety signals have been shown to be especially effective sources of information when the facial information is ambiguous (Zhou and Chen, 2009) or incongruent (Pause et al., 2004). Because in the present study, the participants perceived all faces as clearly negative and anxiety-inducing (individual screening session), the congruent chemosensory information did not add any new information relevant to accomplish the emotion regulation task and might therefore have had no influence. This would imply that visual and chemosensory communication channels constitute specialized independent communication systems, integrating only under circumstances of perceptual uncertainty, or when further information is needed. Thus, the current results suggest that salient visual foreground information can be affected by top–down neuronal control and that contextual chemosensory anxiety cues do alter the general emotional significance of this visual information rather than interacting directly with top–down control mechanisms.

However, the present results leave open the question of whether the effects found in Experiment I have their foundation in earlier processes of stimulus perception, like the structural encoding of the faces (N170), or the allocation of attention (N1/P1) toward the faces. To determine this, in Experiment II an EEG was recorded and the impact of the chemosensory context stimuli (anxiety, sport control, pure cotton pad) was evaluated on early automatic structural encoding of (N1/P1, N170) and late motivational (LPP) attention allocation toward the stimuli.

## Experiment II

### Methods

#### Participants

Thirty-six non-smoking female students (different from those in Experiment I) were classified (according to the SIAS) as either non-socially anxious (LSA, scores < 16, *n* = 18, *M* = 11.61; *SD* = 3.36) or high-socially anxious (HSA, scores > 22, *n* = 18; *M* = 31.22; *SD* = 8.32; group comparison: *p* < 0.001). The mean score of the HSA participants was above the suggested cut-off of 30 for social phobia (Stangier et al., 1999). All participants reported a regular menstrual cycle. Out of the 36 participants, 16 (*N* = 8 HSA) reported to use hormonal contraceptives. All reported that they used no medication, suffered from no mental and physical diseases or general hyposmia. All participants scored low on social, desirability (<5 on the Lie scale of the EPI, Eggert and Ratschinski, 1983), supporting the validity of the self-report data. HSA participants scored within normal range for trait anxiety (STAI) and depressed feelings (DS), while LSA participants scored low on both questionnaires (STAI: *M* = 35.50; *SD* = 5.23, DS: *M* = 5.44, *SD* = 2.81, group difference for both questionnaires: *p* < 0.001). Both groups scored within the medium range for the frequency of everyday-life use of reappraisal (ERQ), and for empathy (SPF). The two groups did not differ in the frequency with which they used reappraisal in everyday life, *p* > 0.20, in empathic feelings, *p* > 0.20 or in age, *p* > 0.20 (*M* = 23.72, *SD* = 4.86, range 19–42). All participants were paid for participation and gave written informed consent. The study was approved by the ethics committee of the DGPs.

#### Stimulus material and stimulus presentation

Chemosensory stimulus material was the same as in Experiment I. In addition, pure cotton pad was introduced as a control stimulus. Prior to usage the pure cotton pads were treated in the same way as the anxiety and sport cotton pads: They were pooled, divided into small portions (1.2 g each) and stored at −20°C. The chemosensory stimuli were presented with a constant-flow (50 ml/s) 5-channel olfactometer and stimuli were presented in three counterbalanced blocks (enhance, down-regulate, watch) of 60 trials each.

The visual stimulus material, consisted of 60 pictures from 30 male actors showing anxious facial expressions with averted gazes to the left and right were chosen from the KDEF set (KDEF, Lundqvist et al., 1997)^2^. The large number of pictures was necessary to prevent habituation effects due to repeated presentation of the face stimuli. Stimulus timing was controlled with the Presentation® software (Version 14, Neurobehavioral Systems, USA).

#### Individual stimulus validation and odor detection session

As in Experiment I, participants were asked to judge the chemosensory stimuli for intensity, pleasantness, unpleasantness and familiarity (10 cm visual analogue scales) during an individual stimulus validation session which took place 7 days prior to the main experiment (see Experiment I section Individual Stimulus Validation and Odor Detection Session for details). Tables 3 and 4 shows participants' stimulus ratings. Chemosensory anxiety signals were perceived as more intense than sport, *t*_(35)_ = 3.38, *p* = 0.002, and cotton pad control stimuli, *t*_(35)_ = 5.15, *p* < 0.001 [main effect stimulus *F*_(2, 68)_ = 13.96, *p* < 0.001, *f* = 0.64]. They were also perceived as more unpleasant than chemosensory sport, *t*_(35)_ = 2.21, *p* = 0.034, and cotton pad control stimuli, *t*_(35)_ = 3.64, *p* = 0.001 [main effect stimulus, *F*_(2, 68)_ = 7.57, *p* = 0.001, *f* = 0.47], and as more familiar than cotton pad control, *t*_(35)_ = 2.72, *p* = 0.010, but not than chemosensory sport stimuli, *t*_(35)_ = 1.85, *p* = 0.073 [main effect stimulus, *F*_(2, 68)_ = 4.57, *p* = 0.015, *f* = 0.37]. Intensity (*p* = 0.068), unpleasantness (*p* = 0.073) and familiarity ratings (*p* = 0.149) between sport and cotton pad control did not differ. There were no differences in pleasantness ratings between any of the stimuli.

**Table 3:** **Mean intensity, pleasantness, unpleasantness, and familiarity ratings of the chemosensory stimuli in Experiment II (EEG)**.

	**Anxiety**		**Sport**		**Cotton pad control**	
	***M***	***SD***	***M***	***SD***	***M***	***SD***
Intensity	4.64	1.68	3.39	1.66	2.72	1.75
Pleasantness	2.61	1.48	2.78	1.64	2.64	2.05
Unpleasantness	3.47	2.35	2.56	1.76	2.03	1.52
Familiarity	3.69	1.95	3.17	2.08	2.64	2.02

*Range 1–9*.

**Table 4:** **Valence and arousal ratings of the chemosensory stimuli in Experiment II (EEG)**.

	**Anxiety**		**Sport**		**Cotton pad control**	
	***M***	***SD***	***M***	***SD***	***M***	***SD***
Valence	−0.81	1.06	−0.36	1.42	−0.11	1.14
Arousal	4.72	1.52	4.81	1.51	4.25	1.50

*SAM Valence range −4–4, SAM Arousal Range 1–9*.

Afterwards, participants specified their feelings of pleasantness and arousal (SAM) in response to the chemosensory stimuli. They rated themselves as feeling more unpleasant (SAM valence) when perceiving the chemosensory anxiety signals compared to cotton pad control stimuli, *t*_(35)_ = 2.50, *p* = 0.017 [main effect stimulus *F*_(2, 68)_ = 3.33, *p* = 0.042, *f* = 0.31]. No more differences were found between HSA and LSA participants concerning the ratings.

Of the participants 19 (53%) were able to differentiate both chemosensory stimuli from cotton pad control (two correct detections for each stimulus within three-alternative forced choice tests including cotton pads from either condition, and two non-used cotton pads, administered via the olfactometer for 2.5 s).

Due to the large number of different facial expressions used in the main experiment (60), individual judgments for the face stimuli were discarded.

#### Experimental session

First electrodes were attached. Then participants received detailed breathing instructions and practiced correct inhalation until they signaled that correct breathing occurred without any effort. Then, stimulus timing was explained and emotion regulation instructions were given. Figure 1 (lower half) shows the stimulus timing. At the beginning of each trial, an anxious facial expression was presented for 1 s to prepare the participant for the upcoming emotion regulation task. Then the written emotion regulation instruction was presented for 1.5 s (enhance, down-regulate, or watch) followed by an exhalation cue. It consisted of a ball decreasing continuously in size across a period of 2.5 s. After the exhalation, the participants started with the inhalation. During the inhalation period (randomly 1–2 s after participants started inhaling), the chemosensory context stimulus was presented for 2.5 s. One second after the onset of the context stimulus the facial expression was presented again for 1.5 s. Participants were instructed to keep inhaling until the end of the picture presentation. During the inter stimulus interval (ISI, duration random between 11 and 13 s), participants rated their current emotional state for valence and arousal (SAM). Mean trial duration was 20 s. After the presentation of 30 trials (10 min) a 5 min break was included. During each block, the 60 facial expressions were presented in random order, and were paired with either a chemosensory anxiety (*n* = 20 trials), sport stimulus (*n* = 20 trials), or cotton pad control (*n* = 20 trials) ^3^. Chemosensory stimuli were equally distributed within blocks, and the same chemosensory stimulus did not occur during more than three consecutive trials.

Participants received the same emotion regulation instructions as in Experiment I. In addition they were told to perceive the stimuli only passively during the watch block. Then they were instructed in how to use emotion regulation instructions during the task. In brief, with onset of the regulation instruction participants had to think about a regulation strategy. With the onset of the target picture they then had to begin regulating and to continue regulating until the onset of the SAM rating scales. Finally, they practiced at least 10 learning trials per experimental condition. The experiment started only after participants signaled full understanding of, and compliance with, the instructions.

#### Data recording

The EEG was recorded with Ag/AgCl electrodes (inner diameter 6 mm) from 25 scalp locations (AF7, FP1, FPz, FP2, AF8, F7, F3, Fz, F4, F8, T7, C3, Cz, C4, T8, P7, P3, Pz, P4, P8, PO7, O1, Oz, O2, PO8) using an electrode cap (EasyCap GmbH, Germany) in reference to the average across all electrodes. In addition both earlobes were recorded. Two electrodes were placed near the right eye (3 cm above, inside the vertical pupil axis and 1.5 cm below, outside the vertical pupil axis) for the recording of vertical and horizontal eye movements. The impedance of the electrodes was kept below 10 kΩ.

The physiological data were recorded, amplified, and filtered with the BrainVision Recorder software (Brain Products GmbH, Munich, Germany) using a sampling rate of 250 Hz, a low-pass filter of 40 Hz (24 dB/octave) and a 50 Hz notch filter. Offline, EEG signals were re-referenced to linked ear lobes and high pass filtered (0.04 Hz, 24 dB/octave), afterwards corrected for eye movements (Gratton et al., 1983) and baseline-corrected (0–200 ms before picture onset). Subsequently, trials contaminated with artifacts (due to sweating, movements, or pronounced alpha-activity: 0.25%) and insufficient inhalation of the chemosensory stimuli (begin of inhalation > 300 ms before picture onset or end of inhalation <700 ms after picture onset: 3.5%) were eliminated. Prior to averaging, in order to ease the component' detection, they were again low-pass filtered (20 Hz, 24 dB/octave).

#### Data analysis

The N1 amplitude was quantified as the maximum peak at frontopolar, frontal and central electrode sites (70–140 ms), the P1 as the maximum peak over parietal and occipital electrode sites (70–140 ms). The N170 amplitude was analyzed as minimum peak over parietal and occipital electrode sites (130–180 ms). The LPP were extracted from all electrodes (LPP mean activity: 400–600 ms).

ERPs were subjected to repeated measure mixed model ANOVA. For the N1 component the ANOVA included the between subject factor *Group* (HSA, LSA) and the within subject factors *Context* (chemosensory anxiety, chemosensory sport, cotton pad control), *Emotion Regulation* (enhance, down-regulate, watch), *Sagittal* electrode sites (frontopolar, frontal, central), and *Transversal* electrode sites (lateral left, left, midline, right, lateral right). For the N170 and the P1 (detected at parietal and occipital sites) the factor *Sagittal* had 2 levels (parietal, occipital), while for the LPP (detected at all electrodes) it had 5 levels (frontopolar, frontal, central, parietal, occipital). For reasons of brevity, effects including electrode factors are presented without follow-up tests.

Mean ratings of valence and arousal were calculated within participants according to the conditions and were subjected to a repeated measures mixed model ANOVA including the between subject factor *Group*, and the within subject factors *Emotion Regulation* (enhance, down-regulate, watch), and *Context* (anxiety, sport, cotton pad control).

Cohen's effect-size *f* was calculated. Huynh–Feldt corrections of degrees of freedom were applied, and corrected *p*-values are reported. Subsequent nested effects (Page et al., 2003) and *t*-tests were calculated. An alpha level of 5% was used for all statistical tests.

### Results

#### Effects of electrode positions

The N1 amplitude was most pronounced at frontopolar and midline electrode sites [main effects for *Transversal*, *F*_(2, 68)_ = 19.58, *p* < 0.001, *f* = 0.76 and *Sagittal*, *F*_(2, 68)_ = 26.67, *p* < 0.001, *f* = 0.89 with largest amplitudes central and frontal midline electrodes [Fz/Cz, interaction *Sagittal* by *Transversal*, *F*_(8, 272)_ = 13.70, *p* < 0.001, *f* = 0.63], while the P1 showed largest amplitudes at parieto-central electrodes [Pz/Oz, interaction *Sagittal* by *Transversal*, *F*_(4, 136)_ = 9.25, *p* = 0.001, *f* = 0.41]. The N170 amplitude was most pronounced at right lateral electrode sites [main effect for *Transversal*, *F*_(4, 136)_ = 22.36, *p* < 0.001, *f* = 0.81], with largest amplitudes were observed over P8 [interaction *Sagittal* by *Transversal*, *F*_(4, 136)_ = 6.92, *p* = 0.001 *f* = 0.45]. Finally, the LPP was most pronounced over parietal and occipital electrode sites [main effect *Sagittal*, *F*_(4, 136)_ = 32.60, *p* < 0.001, *f* = 0.98], and central and right electrode sites [main effect for *Transversal*, *F*_(4, 136)_ = 16.02, *p* < 0.001, *f* = 0.69] with largest potentials over Pz and Oz [interaction *Sagittal* by *Transversal*, *F*_(16, 544)_ = 7.86, *p* < 0.001, *f* = 0.48].

#### Effects of the chemosensory context

***Ratings.*** Participants reported feeling more aroused when the faces were presented in the context of chemosensory anxiety signals (*M* = 5.17, *SD* = 1.14), *t*_(35)_ = 2.19, *p* = 0.035, and chemosensory sport (*M* = 5.08, *SD* = 1.01), *t*_(35)_ = 2.35, *p* = 0.024, compared to the cotton pad control stimuli (*M* = 4.98, *SD* = 0.98). Arousal ratings between faces presented in the context of chemosensory anxiety signals and sport stimuli did not differ, *p* > 0.10 [main effect for *Context*, *F*_(2, 68)_ = 3.83, *p* = 0.041, *f* = 0.34] (Table 5).

**Table 5:** **Emotion regulation effects on subjective ratings for anxious faces in the context of chemosensory anxiety, sport-control or cotton pad stimuli in Experiment II (EEG)**.

	**Cotton pad**			**Sport**			**Anxiety**		
	**Enhance**	**Down-regulate**	**Watch**	**Enhance**	**Down-regulate**	**Watch**	**Enhance**	**Down-regulate**	**Watch**
	***M*_(*SD*)_**	***M*_(*SD*)_**	***M*_(*SD*)_**	***M*_(*SD*)_**	***M*_(*SD*)_**	***M*_(*SD*)_**	***M*_(*SD*)_**	***M*_(*SD*)_**	***M*_(*SD*)_**
SAM_valence_	−1.29_(0.72)_	−0.43_(0.72)_	−0.65_(0.50)_	−1.31_(0.78)_	−0.49_(0.68)_	−0.74_(0.55)_	−1.29_(0.98)_	−0.42_(0.74)_	−0.81_(0.79)_
SAM_arousal_	5.75_(1.06)_	4.32_(1.20)_	4.88_(1.27)_	5.79_(1.12)_	4.46_(1.23)_	4.99_(1.24)_	5.92_(1.19)_	4.49_(1.32)_	5.10_(1.36)_

*SAM_valence_ range: −4–4; SAM_arousal_ range: 1–9*.

**Figure 3:**
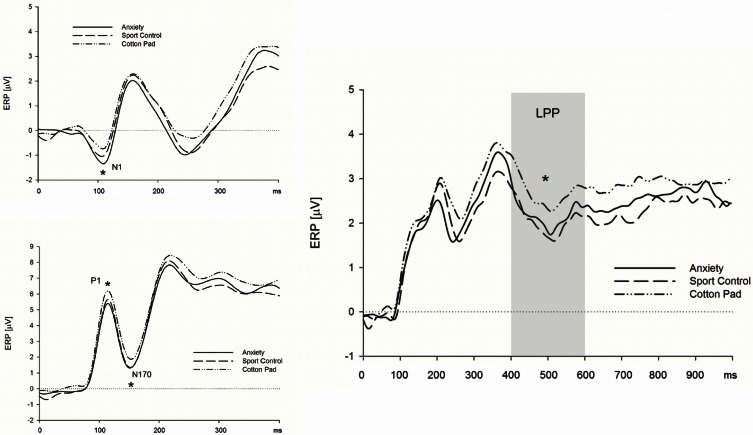
**Effects of chemosensory context on the N1 (upper left), P1 and N170 (lower left) and LPP (right) potential**. Faces presented in the context of chemosensory signals elicited larger N1 and P1 potentials (central scalp locations), and larger N170 potentials (occipital and parietal scalp locations), but smaller LPPs (recorded at all electrode locations). ^*^N1 = 0.041; ^*^P1 = 0.004; ^*^N170 = 0.046; ^*^LPP = 0.009.

***Early ERP components (N1/P1 and N170).*** Above central electrodes, the N1 appeared with larger amplitudes in response to faces presented in the context of chemosensory anxiety signals, *t*_(35)_ = 2.71, *p* = 0.010, and sport stimuli, *t*_(35)_ = 1.99, *p* = 0.054 as compared to faces presented in the context of control stimuli (Figure 3) [interaction *Sagittal* by *Context*, *F*_(4, 136)_ = 2.99, *p* = 0.041, *f* = 0.30, nested effects, context within central electrode sites, *F*_(2, 68)_ = 5.54, *p* = 0.008, *f* = 0.40]. N1 amplitudes for faces presented in the context of anxiety or sport signals did not differ, *p* > 0.10. The P1 amplitudes were larger in the context of chemosensory anxiety (*p* = 0.005) and chemosensory sport stimuli (*p* = 0.006) as compared to faces presented in the context of control stimuli [main effect for *Context*, *F*_(2, 68)_ = 6.02, *p* = 0.004, *f* = 0.36 (Figure 3)]. Amplitudes for faces presented in the context of anxiety or sport signals did not differ, *p* > 0.10. Similar to the N1/P1 deflections, N170 amplitudes were larger for faces presented in the context of chemosensory anxiety, *t*_(35)_ = 2.38, *p* = 0.023, and sport signals, *t*_(35)_ = 2.04, *p* = 0.049 as compared to faces presented in the context of control stimuli [Figure 3, main effect for *Context*, *F*_(2, 68)_ = 3.21, *p* = 0.046, *f* = 0.31]. Amplitudes for faces presented in the context of anxiety or sport signals did not differ, *p* > 0.10 (Figure 3).

***Late positive potential.*** The LPP was larger for faces presented in the context of control stimuli, as compared to those presented alongside with anxiety signals, *t*_(35)_ = 2.33, *p* = 0.026, and sport stimuli, *t*_(35)_ = 2.96, *p* = 0.006. [Figure 3, main effect for *Context*, *F*_(2, 68)_ = 5.04, *p* = 0.009, *f* = 0.38]. The LPP did not differ between faces presented in the context of anxiety signals and sport stimuli, *p* > 0.10 (Figure 3).

#### Effects of social anxiety

**Figure 4:**
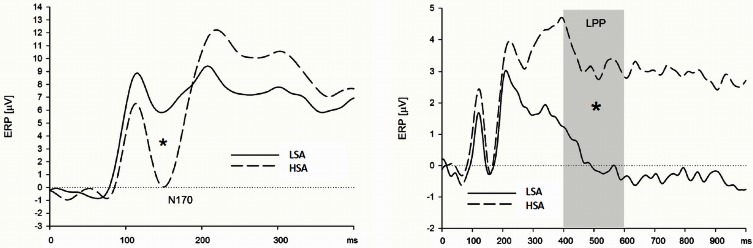
**High socially anxious individuals showed larger N170 (left) and LPPs (right) in response to anxious facial expressions presented without a chemosensory context**. Note that the N170 effect was located in its maximum over left parietal and occipital electrodes positions and the LPP effect was maximal at right lateral electrode positions. ^*^N170 = 0.024; ^*^LPP = 0.003.

***Early ERP components (N1/P1 and N170).*** HSA participants showed larger N170 amplitudes than LSA participants, when they were instructed to watch and to down-regulate their emotions, viewing faces in the context of cotton pad control stimuli (see Figure 4), at left, and midline electrode sites [interaction *Group* by *Transversal* by *Emotion Regulation* by *Context*, *F*_(16, 544)_ = 2.45, *p* = 0.007, *f* = 0.27, nested effects: *Group* by *Emotion Regulation* by *Transversal* within cotton pad *Context*, *F*_(8, 272)_ = 2.66, *p* = 0.024, *f* = 0.28, *Group* by *Transversal* within watch, *F*_(4, 136)_ = 3.66, *p* = 0.031, *f* = 0.33, *Group* within left electrode sites within watch, *F*_(1, 34)_ = 5.91, *p* = 0.021, *f* = 0.42, *Group* within midline electrode sites within watch, *F*_(1, 34)_ = 4.88, *p* = 0.034, *f* = 0.38, *Group* by *Transversal* within down-regulate, *F*_(4, 136)_ = 3.90, *p* = 0.026, *f* = 0.34, *Group* within central electrode sites within down-regulate, *F*_(1, 34)_ = 5.18, *p* = 0.029, *f* = 0.39]. During the enhance condition, there were no differences between HSA and LSA participants, *p* > 0.10. There were no differences between HSA and LSA participants concerning the N1/P1 component.

**Figure 5:**
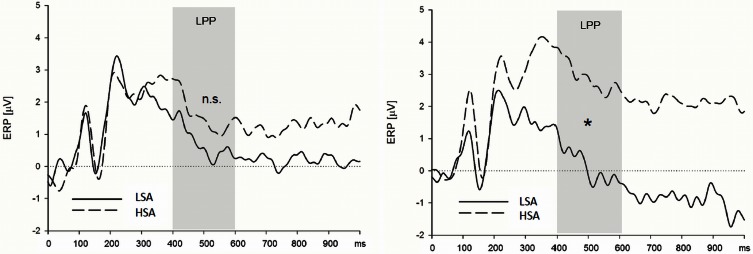
**High socially anxious individuals (HSA) showed larger LPPs in response to faces presented in the context of chemosensory anxiety signals (right side) than did low-socially anxious individuals (LSA)**. There were no differences between HSA and LSA participants for faces presented in the context of chemosensory sport stimuli (left side). Note that LPP effects were maximal over left lateral electrode positions. ^*^LPP right side = 0.001.

***Late positive potential.*** HSA participants showed larger LPPs than LSA participants in the context of the chemosensory control and the chemosensory anxiety stimuli: When the facial expressions were presented in the context of the cotton pad control stimuli, HSA participants showed larger LPPs in the watch, and as a trend, in the down-regulate condition than LSA participants [Figure 4, interaction *Group* by *Emotion Regulation* by *Context* by *Transversal*, *F*_(16, 544)_ = 1.90, *p* = 0.066, *f* = 0.24, nested effects: *Group* within right lateral electrode sites within watch, *F*_(1, 34)_ = 9.87, *p* = 0.003, *f* = 0.54, *Group* within right lateral electrode sites within down-regulate, *F*_(1, 34)_ = 3.60, *p* = 0.066, *f* = 0.33]. In addition, HSA participants showed larger LPPs during the watch (HSA: *M* = 2.89, *SD* = 2.41; LSA: *M* = 0.13, *SD* = 2.15), and the enhance condition (HSA: *M* = 2.90, *SD* = 3.10; LSA: *M* = 0.60, *SD* = 2.97) toward anxious facial expressions in the context of chemosensory anxiety signals [Figure 5, nested effects: *Group* by *Emotion Regulation* by *Context* within right lateral electrode sites, *F*_(4, 136)_ = 3.98, *p* = 0.004, *f* = 0.34, *Group* by *Context* within watch within right lateral electrode sites, *F*_(2, 68)_ = 5.81, *p* = 0.005, *f* = 0.41, *Group* within watch within chemosensory anxiety within lateral right electrode sites, *F*_(1, 34)_ = 13.17, *p* = 0.001, *f* = 0.62, *Group* by *Context* within enhance within lateral right electrode sites, *F*_(2, 68)_ = 5.42, *p* = 0.007, *f* = 0.40, *Group* within enhance within chemosensory anxiety within right lateral electrode sites, *F*_(1, 34)_ = 5.19, *p* = 0.029, *f* = 0.39].

#### Effects of emotion regulation

***Ratings.*** Concerning the valence ratings, all participants described themselves as feeling less negative during the down-regulate [LSA: *t*_(17)_ = 5.31, *p* < 0.001, HSA: *t*_(17)_ = 2.42, *p* = 0.027] and during the watch condition [LSA: *t*_(17)_ = 2.17, *p* = 0.045, HSA: *t*_(17)_ = 3.04, *p* = 0.007] than during the enhance condition [main effect *Emotion Regulation*, *F*_(2, 68)_ = 24.53, *p* < 0.001, *f* = 0.85] (for descriptive statistics see Table 5). However, when down regulating their emotion LSA [*t*_(17)_ = 2.17, *p* = 0.045] but not HSA participants (*p* > 0.10) described themselves as feeling less negative as compared to the watch condition [interaction *Emotion Regulation* by *Group F*_(2, 68)_ = 3.68, *p* < 0.043, *f* = 0.33].

Concerning the arousal ratings, all participants described themselves feeling less aroused during the down-regulate [LSA: *t*_(17)_ = 6.28, *p* < 0.001, HSA: *t*_(17)_ = 3.97, *p* < 0.001] and during the watch condition [LSA: *t*_(17)_ = 3.71, *p* = 0.002, HSA: *t*_(17)_ = 3.12, *p* = 0.006] than during the enhance condition [main effect *Emotion Regulation*, *F*_(2, 68)_ = 36.52, *p* < 0.001, *f* = 1.04]. Like for self-reported valence, when down regulating their emotion LSA, *t*_(17)_ = 5.16, *p* < 0.001, but not HSA participants, *p* > 0.10, described themselves as feeling less aroused than in the watch condition [interaction *Emotion Regulation* by *Group*, *F*_(2, 68)_ = 3.87, *p* < 0.027, *f* = 0.34].

***Early ERP components (N1 and N170).*** Participants showed larger N1 amplitudes when they were instructed to enhance (*M* = −3.36, *SD* = 1.71), as compared to the instruction to down-regulate their emotions (*M* = −2.90, *SD* = 1.51), *t*_(35) = 2.40_, *p* = 0.022 [main effect Emotion Regulation, *F*_(2, 68)_ = 3.00, *p* = 0.056, *f* = 0.81]. Amplitudes did not differ between the enhance- and the watch condition (*M* = −3.29, 2.21), *p* > 0.10, and between the down-regulate and watch condition, *p* = 0.073. There were no effects of emotion regulation on the N170 component.

***Late positive potential.*** Because previous studies show emotion regulation effects mainly for the LPP, the interaction *Group* by *Emotion Regulation* by *Context* by *Transversal*, which was significant as a trend, *F*_(16, 544)_ = 1.90, *p* = 0.066, *f* = 0.24, was further explored. Results indicate that the LPP in response to the faces varied with emotion regulation instruction in LSA participants only. LSA participants showed larger LPPs when they were instructed to enhance their emotion elicited by faces presented in the context of the cotton pad stimuli, as (*M* = 2.28, *SD* = 3.14) than in the watch condition (*M* = 0.144, *SD* = 2.39) in lateral right electrode sites, *t*_(17)_ = 2.51, *p* = 0.023. [nested effects for interaction *Group* by *Emotion Regulation* by *Context* by *Transversal*: interaction *Group* by *Emotion Regulation* by *Transversal* within cotton pad context, *F*_(8, 272)_ = 3.07, *p* = 0.016., *f* = 0.30, *Group* by *Emotion Regulation* within *Transversal*, *F*_(2, 68)_ = 5.03, *p* = 0.009, *f* = 0.38, *Emotion Regulation* within right electrode sites within LSA participants, *F*_(2, 68)_ = 4.43, *p* = 0.015, *f* = 0.36, *Emotion Regulation* within right electrode sites within HSA participants, *p* > 0.10]. There were no differences between the enhance and down-regulate (*M* = 1.26, *SD* = 2.35), *p* > 0.10, and between the watch and down-regulate conditions, *p* = 0.098.

## General discussion

Two experiments investigated withdrawal related motor behavior (Experiment I) and ERP correlates (Experiment II) of perception and regulation of facial expressions presented in the context of human chemosensory signals (sport, anxiety) in a group of HSA and a group of LSA individuals. In Experiment I, regardless of emotion regulation or social anxiety, startle responses and emotion regulation effects toward faces presented in the context of chemosensory sport or anxiety stimuli did not differ from each other, suggesting a more powerful influence of the salient visual foreground information as compared to the contextual chemosensory cues on withdrawal related motor behavior. However, although presented at threshold level, HSA individuals as compared to LSA individuals showed a hyperreactivity in withdrawal related motor behavior in the context of chemosensory anxiety signals.

In line with the results of Experiment I, Experiment II also found a preferential processing of contextual chemosensory sport and anxiety information, as indexed by reduced elaborative processing (LPP), compared to the cotton pad control stimuli. It has already been shown that olfactory and visual information are integrated on a neuronal level (Gottfried and Dolan, 2003), and cross-modal integration has been demonstrated for chemosensory signals and facial expressions (Pause et al., 2004; Zhou and Chen, 2009). Moreover, a recent study demonstrated that the perception of chemosensory information (sport/anxiety), elicits large P3 amplitudes (Pause et al., 2010), suggesting that the processing of this information depends on the allocation of additional neuronal resources. Thus, the additional chemosensory anxiety context information in the present study most likely have distracted neuronal resources from the elaborative processing of the concurrently presented facial expressions, leading to reduced late ERPs toward the faces. This data are consistent with previous reports showing preferential processing of olfactory information in a direct contrast with visual stimuli (Royet et al., 2000; Adolph and Pause, 2012), and reports showing the importance of chemosensory information for social interaction (McClintock, 1971; Kaitz et al., 1987; Wedekind and Füri, 1997; Jacob et al., 2002; Preti et al., 2003). Consistently with the literature, this suggests that the contextual chemosensory information is processed preferentially.

Interestingly, in contrast to the results for late ERPs, larger early (N1/P1 and N170) ERPs for facial expressions were found when they were presented in a social chemosensory context, suggesting an enhancement of early stimulus processing stages for the faces by human chemosensory signals. The present data are in accord with previous research showing enhanced P1 amplitudes for fearful and angry faces as compared to neutral faces (e.g., Batty and Taylor, 2003; Kolassa and Miltner, 2006). Because the P1 has been shown to be attention-sensitive (see Mangun, 1995), the current data suggest that the faces presented in context of chemosensory stimuli received more attentional processing than those presented without a chemosensory context. Interestingly, in a recent study, participants showed faster response times and larger P1 amplitudes toward visual stimuli presented at a location previously cued with emotional prosody (Brosch et al., 2009). The current results extend these findings and show also that human chemosensory signals can enhance early perceptual processing of concurrently presented visual stimuli. Thus, the present and previous data suggest that emotional context information serves to guide perceptual processing, probably through initiation of early attention-associated processes, leading to higher vigilance toward concurrently presented stimuli.

Ratings indicate that the chemosensory anxiety signals were perceived as more intense, unpleasant, and familiar as the sport signals and as cotton pad control. Therefore, it cannot be completely ruled out that some of the observed effects on ERPs occurred because the context stimuli were perceived differently. However, overall, the chemosensory stimuli were described as low in intensity, and as only mildly unpleasant. The subjective emotional responses toward them were described as rather neutral. Furthermore, while differences in ERP effects were observed for anxiety and sport signals in comparison to the cotton pad control stimuli, differences in subjective ratings were evident for anxiety in comparison to the sport and the cotton pad control stimuli. Finally, in line with previous reports, the effects of chemosensory stimuli occurred largely independently of conscious stimulus processing. Only 50% of the participants were able to consciously distinguish the chemosensory stimuli from cotton pad control. Moreover, differences in stimulus ratings were observed between the chemosensory anxiety and both control stimuli (i.e., cotton pad and chemosensory sport stimuli) only. In contrast, differences in ERPs were observed between cotton pad control and the two chemosensory stimuli (i.e., anxiety, sport), but not between the cotton pad control and the chemosensory sport stimuli. Thus, taken together it seems unlikely that the observed ERP effects are due to the differences in the cognitive evaluation of the chemosensory stimuli.

Enhanced LPPs in socially anxious individuals are found for faces presented in the context of chemosensory anxiety signals. In line with this, HSA participants exhibited larger withdrawal related motor behavior in response to chemosensory anxiety signals than did LSA participants (Pause et al., 2009). In addition, HSA participants compared to LSA participants showed enhanced neuronal processing of the fearful expressions presented without a chemosensory context. This is reflected in enhanced early (N170) and late (LPP) ERPs in HSA participants. Previous studies have shown enhanced automatic guidance of motivated attention (Schupp et al., 2004) toward fearful faces in social anxiety (Mühlberger et al., 2009), and socially anxious individuals have been shown to respond to angry or fearful faces with increased amygdala activation (Straube et al., 2004; Phan et al., 2006).

Our results extend these findings and suggest that even components related to the early structural encoding (N170) of fearful facial expressions are enhanced in socially anxious individuals. In addition, the observed enhanced LPPs in HSA participants indicate an enhanced elaborative processing of fearful facial expressions compared to LSA participants (see also Kolassa and Miltner, 2006; Moser et al., 2008). Thus, converging evidence from previous research and the current study suggest a general processing bias in favor of threatening (angry, fearful) faces and chemosensory signals of anxiety in socially anxious participants, as indexed by deviant stimulus processing during late elaborative and early processing stages.

Correspondingly, no emotion regulation effects on the LPP were found for HSA participants in response to the fearful facial expressions. Maybe this is reflected in a ceiling effect of emotional engagement in HSA participants toward fearful faces that could not be altered using cognitive emotion regulation. This assumption is also supported by the fact that HSA participants showed large LPPs during the watch and the down-regulate conditions compared to LSA participants. These findings provide evidence that during emotion regulation, motivational attention (as reflected by the LPP) is deficient in social anxiety disorders. This is important in terms of theories focusing on attentional biases in social phobia: The reduced ability to distract attention from the feared stimulus might be one source of the attentional biases found frequently in social phobia.

Interestingly, as revealed in Experiment I, despite their hyperreactivity toward the faces presented in the anxiety context, and consistent with previous reports (Goldin et al., 2009) we found no evidence for impaired emotion regulation of the startle response in HSA participants. These results indicate a dissociation of the impact of emotion regulation on early attention related visual stimulus processing stages (Experiment II) and on the initiation of behavioral action tendencies (Experiment I). Thus, socially anxious individuals, although impaired in voluntarily regulating motivated attention toward fear relevant stimuli, are not impaired in the later regulation of withdrawal related action tendencies. Interestingly, no differences were found in the self-reported frequency of use of regulation strategies in everyday live and in the post experimental questionnaire between HSA and LSA participants. Thus, because they are frequently confronted with their feared situation, HSA participants may have simply developed more effective regulation strategies and thus are able to overcome their initial hyperreactivity toward the social cues in the present study. Indeed, initial evidence suggest that social phobics show less signal change in emotion regulation related brain areas during cognitive reappraisal, but show no impairment in emotion regulation outcome (Goldin et al., 2009) suggesting that comparable emotion regulation outcome to that of healthy controls is accompanied with the allocation of fewer neuronal resources in socially anxious individuals.

The current findings extend the existing literature and show that socially anxious individuals have a processing bias (Hirsch and Clark, 2004) not only toward visual social signals of threat (Merckelbach et al., 1989; Stein et al., 2002; Straube et al., 2004; Kolassa and Miltner, 2006; Phan et al., 2006; Blair et al., 2008; Moser et al., 2008; Mühlberger et al., 2009), but also in response to social chemosensory signals of anxiety. This processing bias involves both early attentional, as well as late behaviorally relevant information processing. Interestingly, initial evidence shows that social anxiety might also be accompanied by an enhanced vigilance toward chemosensory signals of aggression/dominance (Adolph et al., 2010). This suggests that the processing bias in social anxiety toward social threat information may be generalized to multiple social communication channels. This view is also supported by findings of increased activation of emotion processing brain areas in social phobics toward threatening (angry) prosody (Quadflieg et al., 2008).

Taken together, findings from the literature and the current results suggest a specific multichannel sensitivity of socially anxious individuals toward threat related social information. These findings have important implications. Etiological models suggest that information-processing biases play a central role for the development and maintenance of the disorder (Clark and Wells, 1995). Specifically, it has been argued that socially anxious individuals fail to habituate during social encounters and exhibit continued subjective distress, which may lead to subsequent avoidance, being implicated in the maintenance of the disorder (Beidel et al., 1985). The observed processing biases toward social threat stimuli, especially in terms of contextual chemosensory information, may in part mediate this failure in habituation to the social situation. This assumption is also supported by the fact that HSA participants compared to LSA participants showed significant larger withdrawal related motor behavior under perceptual uncertainty (Experiment I), that is, briefly after stimulus offset. This suggests a sustained hypervigilance and hyperreactivity even after the threatening situation is over. Moreover, in HSA individuals the startle responses in the context of chemosensory anxiety signals did not habituate within the trials, while they did so for LSA individuals. Thus, therapeutic interventions may profit from incorporating chemosensory, visual, and acoustic threat signals into therapeutic treatments.

As in previous studies, results show that LSA participants could successfully regulate their emotions, as indicated by their ratings of emotional experience, and by the LPP results. In detail, participants rated themselves to feel less negative, and less aroused when down regulating their emotions, while they described themselves as feeling more negative and more aroused when enhancing their emotions. In line with previous reports (Moser et al., 2009), is that the LPP was larger when LSA participants were instructed to enhance their emotions in response to anxious expressions presented in the context of control stimuli (cotton pad), as compared to the watch condition, indicating effective enhancement of emotional responses to fearful facial expressions. We did not find the expected reduction of the LPP in the down regulation condition, as reported previously (Hajcak and Nieuwenhuis, 2006; Moser et al., 2006, 2009). This could be due to the nature of the facial stimuli. Emotional facial expressions are often described as only mildly arousing (Britton et al., 2006; Alpers et al., 2011) as compared to the highly arousing emotional scenes used in other emotion regulation studies. Moreover during the experiment participants were confronted with a large number of trials and thus habituation of emotional responses cannot be ruled out. This may have caused a rather low emotional involvement of the LSA participants, leading to the present null results for the down regulation condition. However, in general, the present results indicate that emotions elicited by threatening social stimuli can be manipulated using cognitive linguistic emotion regulation strategies.

Within the N1 latency range ERPs were larger during the instruction to enhance than to down-regulate emotions. The N1 component is especially sensitive to selective attention (Hillyard et al., 1998). Interestingly, results from a recent emotion regulation study using eye tracking show that selective attention was controlled by the participants differently depending on whether the regulatory goal was to decrease or increase emotions (Van Reekum et al., 2007), suggesting that in the present study attention may have been allocated automatically, i.e., without conscious control, depending on the regulatory goal. In contrast to the N1 results, the face-specific N170 component was not affected by emotion regulation. Early responses at central scalp locations (N1 in the present study) index general aspects of selective attention, while ERPs in the latency range of the N170 reflect modality-specific processing stages (Van Voorhis and Hillyard, 1977). Thus, the results observed for the N170 and the N1 in the present study may arise from distinct aspects of perceptual stimulus processing, and suggest that the structural encoding of facial expressions (N170) may not necessarily rely on the allocation of attentional resources. Taken together, the present study shows that also ERPs as early as the N1 are affected by emotion regulation. Furthermore, they support the view that attention selection may be a frequently used emotion regulation strategy in everyday life (Gross et al., 2006). The data from Experiment II are also in line with the findings that withdrawal related motor behavior toward faces in the context of chemosensory signals can be regulated successfully. Thus, in terms of emotion regulation, the present study extends previous reports showing that cognitive linguistic emotion regulation strategies are generally useful in regulating visual and olfactory emotional cues, and shows that early attention related stimulus processing and motor behavior toward chemosensory context dues can be regulated effectively.

One shortcoming of the present study could be that we did not assess the basic emotions elicited by the chemosensory stimuli in the perceivers. Therefore, we cannot completely rule out the possibility that at least some of the participants might have experienced disgust while perceiving the stimuli. However, across studies it has been shown that in general emotional reactions to chemosensory stimuli are rather weak and that people cannot label the stimuli in terms of basic emotions, probably because of rather low detection rates of the chemosensory stimuli (for an overview see Pause, 2012). However, although the effect was rather weak, one study has shown that when participants are asked to decide which of the 6 basic emotions fits best to the emotional state of the donors, chemosensory anxiety stimuli are more often described to smell anxiety-like (Pause et al., 2009). Thus, on basis of the literature, it seems rather unlikely that the participants experienced disgust in the present study. Additionally, the data show that participants described their emotions as only mildly unpleasant (*M* = −0.6, on a scale ranging from −4 = very unpleasant to +4 = very pleasant) and these ratings did not differ between the chemosensory conditions, suggesting only very little emotional involvement at all.

Finally, because only fearful facial expressions were used in the present study, only a main effect of chemosensory context could be tested, not an interaction between facial expression and chemosensory context. Thus, future studies should include neutral facial expressions to address the question of differential effects of chemosensory context on the processing of emotional and neutral facial expressions (for example on N1/P1 or N170 amplitudes).

## Conclusion

In sum, the present results show that social chemosensory information constitute powerful context cues. They are capable of altering the processing of emotional facial expressions in guiding motivated attention and in altering withdrawal related motor behavior. Moreover, the present study shows for the first time that socially anxious individuals at risk for social phobia are especially sensitive toward contextual social chemosensory anxiety signals. They show enhanced withdrawal related motor behavior, no habituation (Study I) and enhanced allocation of attention toward faces presented in the context of these signals as compared to the low-socially anxious individuals. An enhanced vigilance and excitability as well as a lack of habituation toward threatening social context cues may form one basis for the processing bias of threatening social information in socially anxious individuals and may thus be an important factor for the maintenance of the disorder. Thus, people suffering from social phobia may profit from incorporating social context cues into therapeutic treatments.

### Conflict of interest statement

The authors declare that the research was conducted in the absence of any commercial or financial relationships that could be construed as a potential conflict of interest.

## Footnotes

^1^In order to obtain homogenous chemosensory stimulus material the sweat samples were collected from males only. We have previously shown that the effects of chemosensory stimuli are independent of the sex of the donor (Pause et al., 2009).

^2^Stimuli with averted gaze were chosen because it has been demonstrated that averted fearful expression elicit more negative affect in the perceiver than fearful expressions with direct gaze (Hess et al., 2007).

^3^The relatively small number of trials per condition was necessary in order to prevent the chemosensory stimuli from habituation.
